# Lymphocyte-activating gene 3 expression in tumor cells predicts immune checkpoint inhibitor response in triple negative breast cancer

**DOI:** 10.3389/fonc.2023.1146934

**Published:** 2023-03-16

**Authors:** Ji-Yeon Kim, Jeehyun Kim, Eun Yoon Cho, Yeon Hee Park, Jin Seok Ahn, Kyoung-Mee Kim, Young-Hyuck Im

**Affiliations:** ^1^ Division of Hematology-Oncology, Department of Medicine, Samsung Medical Center, Sungkyunkwan University School of Medicine, Seoul, Republic of Korea; ^2^ Biomedical Research Institute, Samsung Medical Center, Sungkyunkwan University School of Medicine, Seoul, Republic of Korea; ^3^ Department of Pathology and Translational Genomics, Samsung Medical Center, Sungkyunkwan University School of Medicine, Seoul, Republic of Korea; ^4^ Samsung Advanced Institute for Health Sciences and Technology, Sungkyunkwan University School of Medicine, Seoul, Republic of Korea

**Keywords:** LAG-3, PD-L1, PD-1, triple negative breast cancer, immune checkpoint inhibitor

## Abstract

**Introduction:**

Immune checkpoint inhibitor (ICI) is one of the standard treatment strategies in triple negative breast cancer (TNBC). However, the benefit of ICI with chemotherapy is limited in metastatic TNBC. In this study, we evaluated the effect of PD-L1 and LAG-3 expression on tissue microenvironment of mTNBC treated with ICI.

**Methods:**

We reviewed representative formalin-fixed paraffin embedded specimens from metastatic or archival tumor tissues of TNBCs who treated with PD-1/PD-L1 inhibitors in metastatic setting. We used the Opal multiplex Detection kit with six antibodies (anti-PD-L1, anti-LAG-3, anti-CD68, anti-panCK, anti-CD8, anti-CD107a/LAMP antibody).

**Results:**

We evaluated the association between LAG-3+cells and survival outcome regarding CK expression. Stromal LAG-3+/CK+ and LAG-3+/CK- cells were not associated with ICI-progression free survival(PFS) (P=0.16). However, LAG-3+ cell distributions in the tumor area impacted on ICI-PFS. A high density of LAG-3+CK+ cells was associated with shorter ICI-PFS compared with low densities of both LAG-3+CK+ and LAG-3+CK- cells (1.9 vs. 3.5 months). In addition, a high density of LAG-3+CK- cells had a relatively longer ICI-PFS compared with other groups (P=0.01). In terms of total area, the pattern of densities of LAG-3+CK+ cells and LAG-3+CK- cells were similar to those in the tumor area In addition, ICI-PFS of LAG-3+CK- and LAG-3+CK+ cell densities in the total area was equal to that in the tumor area.

**Discussion:**

In conclusion, our findings revealed tumor-intrinsic LAG-3 expression was the resistance mechanism toward PD-1/PD-L1 inhibitors in mTNBCs. Multivariate analysis also suggested that LAG-3 expression in tumor cells was an independent predictive biomarker.

## Introduction

Triple negative breast cancer (TNBC), defined as estrogen receptor (ER)-, progesterone receptor (PgR)-, and human epidermal growth factor receptor 2 (HER2)-negative breast cancer (BC) has poor prognosis compared to other BC subtypes (1). In addition, effective targeted agents for TNBC are rare, and traditional cytotoxic chemotherapy has been the basis of treatment for metastatic TNBC (mTNBC) (2).

To date, immune checkpoint inhibitor (ICI) is one of the standard treatment strategies in TNBC. A phase II clinical trial of pembrolizumab, an anti-PD-L1 antibody, monotherapy revealed the therapeutic potential of PD-L1-positive mTNBC (3). KEYNOTE-355, a phase III study of pembrolizumab with cytotoxic chemotherapy, showed better survival after combination treatment in PD-L1-positive mTNBC as the first-line treatment compared to cytotoxic chemotherapy only (4). In addition, atezolizumab and anti-PD-L1 inhibitor with nab-paclitaxel have also shown efficacy as a first-line treatment in PD-L1-positive TNBC in phase III IMpassion in 130 clinical trial (5). However, the benefit of ICI with chemotherapy is limited in mTNBC, and studies to find predictive biomarkers and resistance mechanisms have been performed.

PD-L1 status and tumor infiltrating lymphocytes (TILs) are well-known predictive biomarkers of ICI (6, 7). The tumor microenvironment (TME), which consists of T cells, macrophages, fibroblasts, and many other cells, was suggested as a predictive biomarker and resistance mechanism of ICI (8). In addition, other immune checkpoint regulators such as indoleamine 2,3-dioxygenase 1 (IDO1), lymphocyte activating gene-3 (LAG-3), and T cell immunoglobulin and mucin domain-containing-3 (TIM-3) have potential as prognostic and predictive biomarkers of mTNBC treated with ICI (9–11).

LAG-3 is a transmembrane protein found on activated T cells and natural killer (NK) cells, where it mainly functions as a receptor that delivers inhibitory signals (12). Recent clinical trials of LAG-3 antibody demonstrated its antitumor activity (13). In addition, a PD-L1/LAG-3-bispecific antibody has been developed as another ICI (14).

In this study, we evaluated the effect of PD-L1 and LAG-3 expression on TME of mTNBC treated with ICI. This study was aimed to identify the role of LAG-3 expression in mTNBC with ICI and to establish the availability of an LAG-3 inhibitor.

## Methods

### Study population

Metastatic TNBC patients who received ICI were enrolled in this analysis. We collected BC tissues regardless of archival or fresh tissue in a metastatic setting. Baseline demographic characteristics, histologic characteristics, and previous treatment history were collected from clinical data. This study was performed in accordance with the principles of the Declaration of Helsinki and the Korean Good Clinical Practice guidelines. Collection of specimens and associated clinical data used in this study was approved by the Institutional Review Board of Samsung Medical Center (IRB File No. 2022-08-122), and we received informed consents for human-derived materials.

### Pathologic preparation

We obtained representative formalin-fixed paraffin embedded (FFPE) specimens from metastatic or archival tumor tissues of TNBCs. Then, we dissected FFPE to a 2 µm thickness and fixed them to coated slides (Bond Plus slides, Leica, Germany). We applied bond RX auto-strainer for de-paraffinization, rehydration, and heat-induced epitope retrieval (HIER). An ER1 (citrate-based pH 6) solution heated at 98°C for 20 minutes was used for HIER condition.

### Automation immunohistochemistry detection

We used the Opal multiplex Detection kit (Akoya, MA, USA) for slide staining based on the manufacturer’s instructions. We used six antibodies and six colored Opal dyes for staining. Anti-PD-L1 antibody (22C3, DAKO) was incubated first, and then horseradish peroxidase (HRP) conjugated secondary antibody (Ms+Rb polymer, Akoya) and Opal dye 570 were attached. Anti-LAG-3 antibody (EPR4392, Abcam), secondary antibody, and Opal dye 520 were conjugated in the second cycle, followed by anti-CD68 antibody (KP1, Novocastra), secondary antibody, and Opal dye 620; anti-panCK antibody (AE1/AE3, DAKO), secondary antibody, and Opal dye 690; and anti-CD8 antibody (SP57, Ventana), secondary antibody, and Opal dye 480. Last, anti-CD107a/LAMP antibody (H4A3, abcam), secondary antibody, TSA-DIG (Akoya), and Opal 780 were conjugated. In the final step, slides were treated with ProLong Gold AntiFade reagent with a DAPI mount (Invitrogen, 50 µl for each slide). After manufacturing, slide analysis was performed using a Vectra Polaris imaging system (Akoya) and inForm software (Version 2.8.0; Akoya).

### Statistical analysis

Correlations between clinical characteristics and tumor response were analyzed by two-sided Student’s t-test and Fisher’s exact test. Evaluations of the median values of protein expression between two groups were performed using independent two sample t-test after Levene’s test.

The response rate (ORR) for ICI was measured using RECIST, version 1.1, and was defined to include patients who achieved complete response (CR) or partial response (PR). The disease control rate (DCR) for ICI was defined as CR, PR, or stable disease (SD). Progression-free survival (PFS) for ICI was defined as the elapsed time from the first date of ICI treatment to detection of disease progression. Overall survival (OS) was defined as the duration between date of diagnosis of metastatic disease and death. Distant Recurrence Free Survival(DRFS) was the duration between initial date of BC diagnosis and the date of distant recurrence. PFS and OS were analyzed using the Kaplan-Meier method. Cox proportional hazard regression was used to estimate hazard ratios (HRs) and 95% confidence intervals (CIs). Two-tailed p-values < 0.05 were considered statistically significant, and IBM SPSS Statistics ver. 21 (IBM Co., Armonk, NY) was used for analysis of all data.

## Results

### Baseline characteristics

In total, 40 mTNBC patients were analyzed (**Supplementary Figure 1**). Clinical characteristics of these patients are described in **Table 1**. The median age at mTNBC diagnosis was 43 (range: 23.5, 64.5) years, and there were three patients with *de novo* disease (7.5%). Germline BRCA state was tested in 32 patients, and 4 (12.5%) harbored the germline BRCA1 mutation. Of 37 recurred BC patients, 90% were treated with anthracycline or taxane. In addition, capecitabine was used in 42.5% patients as an adjuvant setting after neoadjuvant chemotherapy. Pembrolizumab was used in 52.5% of mTNBC patients, and the remaining were treated with atezolizumab. ICI as the first-line treatment was used in 57.5% of patients, and 22.5% of patients received ICI after a third line of treatment in a metastatic setting.

**Table 1:** Baseline clinical and pathologic characteristics (N=40).

Characteristics	N (%)	Characteristics	N (%)
Age (median, range)	43.0 (23.5,64.5)	Previous treatment	n=37
Menopausal status		Neoadjuvant CTx ^1^	29 (72.5)
Pre-menopause	33 (72.5)	Adjuvant CTx	26 (65.0)
Post-menopause	7 (17.5)	Adjuvant RTx^2^	31 (77.5)
BC^3^ status		Chemotherapy	n=37
Recurred	37 (92.5)	Anthracycline	36 (90.0)
*De novo*	3 (7.5)	Taxane	36 (90.0)
Nuclear grade		Capecitabine	17 (42.5)
2	13 (32.5)	Platinum	12 (30.0)
3	19 (47.5)	Distant recurrence-free interval n=37	
Unknown	8 (20.0)	<24 months	28 (75.7)
Histologic grade		>24 months	9 (24.3)
2	11 (27.5)	Line of previous CTx^1^ in a metastatic setting	
3	21 (52.5)	0	23 (57.5)
Unknown	8 (20.0)	1	5 (12.5)
Tissue organ		2	3 (7.5)
Breast	21 (52.5)	3 or more	9 (22.5)
Skin	5 (12.5)	Germline BRCA status	
Brain	4 (10.0)	Negative	28 (70.0)
Liver	3 (7.5)	Pathologic variant	4 (10.0)
Lymph node	2 (5.0)	Unknown	8 (20.0)
Ovary	2 (5.0)	Immune checkpoint inhibitor	
Lung	1 (2.5)	Pembrolizumab	21 (52.5)
Pleura	1 (2.5)	Atezolizumab	19 (47.5)
Peritoneum	1 (2.5)	Tissue status	
		Archival	16 (40.0)
		Metastatic	24 (60.0)

1: Chemotherapy; 2: Radiotherapy; 3: Breast cancer.

In terms of tissue status, 40% were archival tissues and 60% were metastatic biopsies. Breast was the most common organ (51.5%), followed by skin (12.5%), brain (10.0%), and liver (7.5%).

### Response to immune checkpoint inhibitor

**Figure 1:**
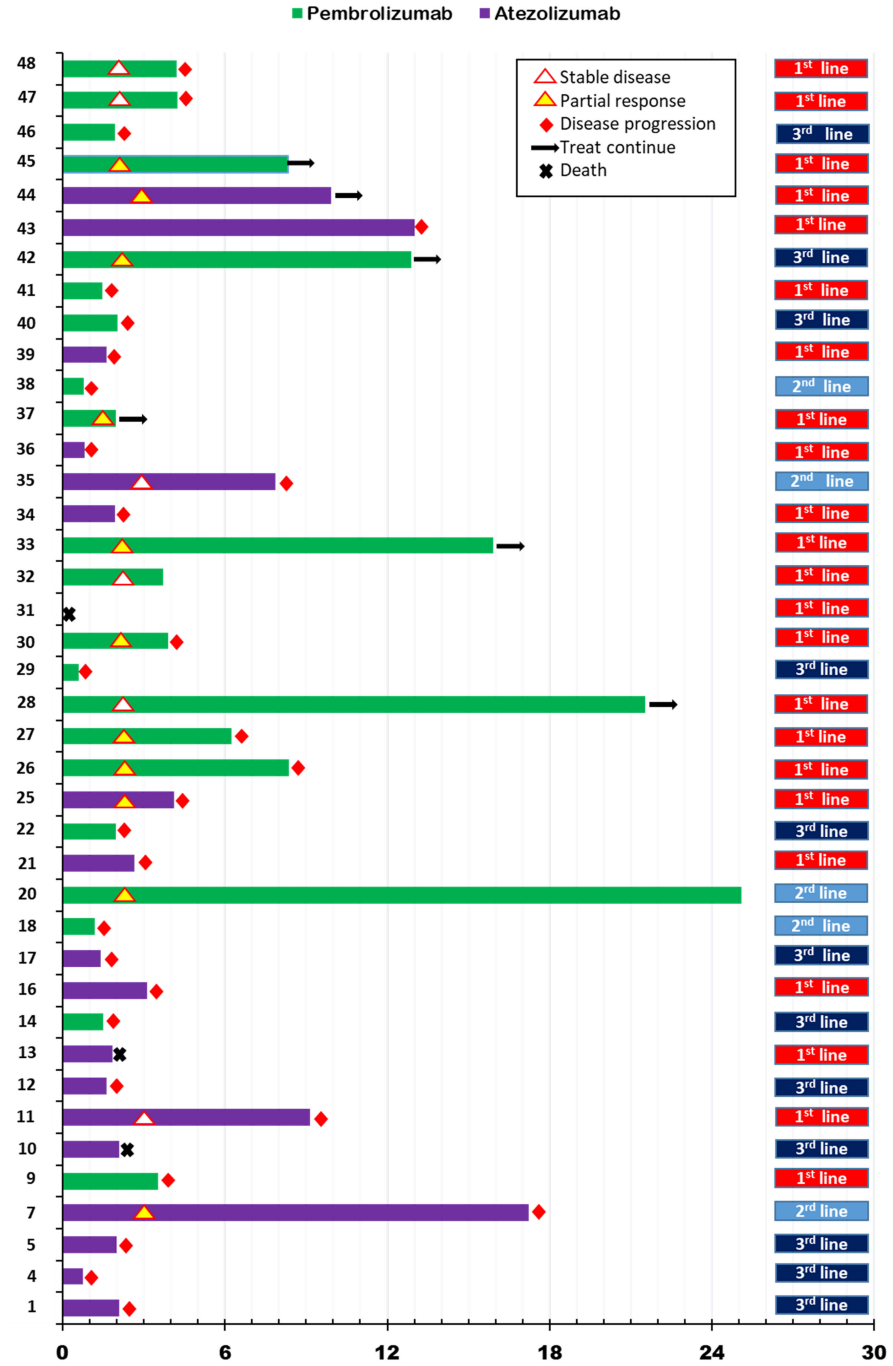
Swimmer’s plot for progression free survival according to immune checkpoint inhibitor treatment in metastatic setting.

We describe the process of ICI treatment for 40 patients in **Figure 1**. Of these patients, tumor assessment was performed in 37. Three patients died due to disease progression without tumor assessment and were classified as progression of disease (PD). The ORR for ICI was 27.5% (11 of 40 patients). No patient achieved CR, although 11 showed PR. In particular, the ORR for atezolizumab was 15.8%, and that for pembrolizumab was 38.1% (p=0.16). With regard to treatment lines, ORR for first-line ICI was 34.8%, that for second-line treatment was 40%, and that of third or additional lines was 8.3% (p=0.16). The DCR for ICI was 42.5%, that for atezolizumab was 19%, and that for pembrolizumab was 57% (p=0.06). A 52% DCR was observed with first-line ICI treatment, 60% DCR with second-line ICI, and 8.3% for the third or additional lines (p=0.02).

In this analysis, the median follow-up duration was 14.1 months (interquartile range [IQR]:7.5, 25.2). Median PFS for ICI (ICI-PFS) was 3.5 months (95% confidence interval [CI]:1.6, 5.5) and median OS was 18.5 (95% CI: 5.6, 31.4) (**Supplementary Figures 2A, B**). PFS for pembrolizumab was 4.2 months compared with 2.7 months for atezolizumab (p=0.34), while the OS for pembrolizumab was 18.5 months and that for atezolizumab was 17.3 months (P=0.64) (**Supplementary Figures 2C, D**). Regarding ICI treatment lines for metastatic disease, the first line showed PFS of 4.2 months, the second line 1.4 months, and the third or additional line 2.0 months (p=0.08) (**Supplementary Figure 2E**).

**Figure 2:**
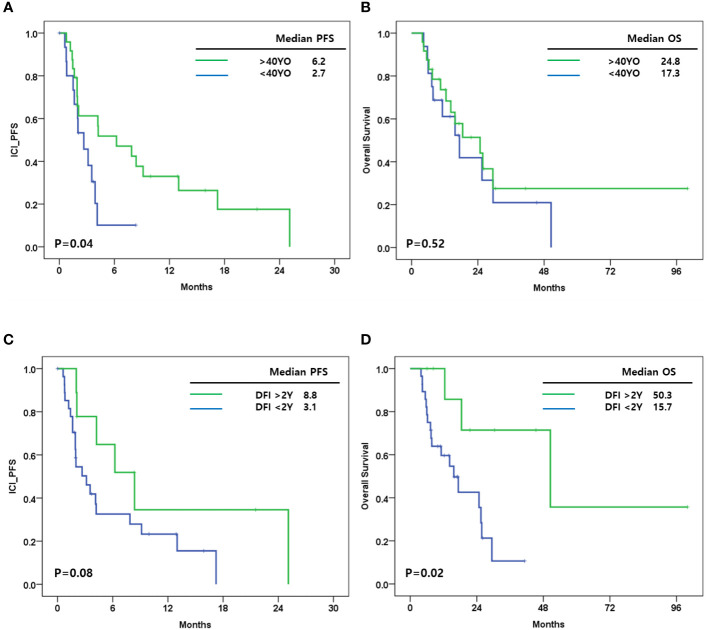
**(A)** Immune checkpoint inhibitor progression free survival (ICI_PFS) according to age at breast cancer diagnosis; **(B)** Overall survival (OS) according to age at breast cancer diagnosis; **(C)** ICI_PFS according to distant recurrence free interval (DRFI); **(D)** OS according to DRFI.

Other clinical factors affecting ICI-PFS and OS were analyzed. Among baseline clinical factors, patients younger than 40 years had shorter ICI-PFS compared with those older than 40 years (2.7 vs. 6.2 months, P=0.04). An OS of 17.3 months for patients younger than 40 was observed compared with 24.8 months for those older than 40 years (p=0.52) (**Figures 2A, B**). In addition, the distant recurrence-free interval (DRFI) affected ICI-PFS and OS (median ICI-PFS [DRFI <24 vs. >24 months]: 3.1 vs. 8.8, P=0.08; median OS [DRFI <24 vs. >24 months]: 15.7 vs. 50.3, p=0.02) (**Figures 2C, D**). Other clinical factors, germline BRCA mutation, and *de novo* disease did not affect PFS and OS (data not shown).

### Expression of six proteins in TNBC

Tumor cell and stromal cell counts and cell densities (cells/mm^2^) were analyzed (**Supplementary Table 1**). All specimens had 1000 or more tumor cells, and stromal cell count was 1000 or more in 36 specimens (90%). We evaluated the six protein markers CK, CD8, CD68, and CD107a by cell type and PD-L1 and LAG-3 as immune checkpoint markers. Among the four cell-type proteins, CK+ cells and CD107a+ cells were more frequently observed in tumor cells compared with stromal cells (p <0.01 and p<0.01). In addition, the densities of CD8+ cells were similar between tumor and stroma (p=0.77). The other cell type markers CD107a+ CD8+ and CD68+CD107a- were similarly distributed in tumor and stroma (p=0.27 and p=0.50) (**Supplementary Table 1**).

For immune checkpoint markers, LAG-3 was more densely expressed in tumor cells compared with stromal cells (p=0.01). LAG-3+/CK+ cells were more frequently observed in tumor cells (p<0.01), while LAG-3+/CK- cells were more populous in stromal cells (p=0.01). Among LAG-3+CK- cells, LAG-3+/CD107a+ were frequently observed in stromal cells (p=0.01), while LAG-3+CD8+ cells were not (p=0.19). PD-L1+ cells were denser in tumor cells compared with stromal cells (p<0.01). However, PD-L1+/CD8+ cell density did not differ in tumor and stroma (p=0.37) (**Supplementary Table 1**; **Supplementary Figure 3**).

### Prognostication value of immune cell distribution

We evaluated the relationship between cell densities in tumor and stroma. In CD8+ cells, cell density in the tumor area was positively correlated with that in stroma (**Supplementary Figure 4A**). CD107a+ cells were more commonly observed in tumors than in stroma (**Supplementary Figure 4B**). In addition, several tissues had no or few CD107a+CD8+ cells in either tumor or stroma, and some tissues had cells in both areas (**Supplementary Figure 4C**).

**Figure 3:**
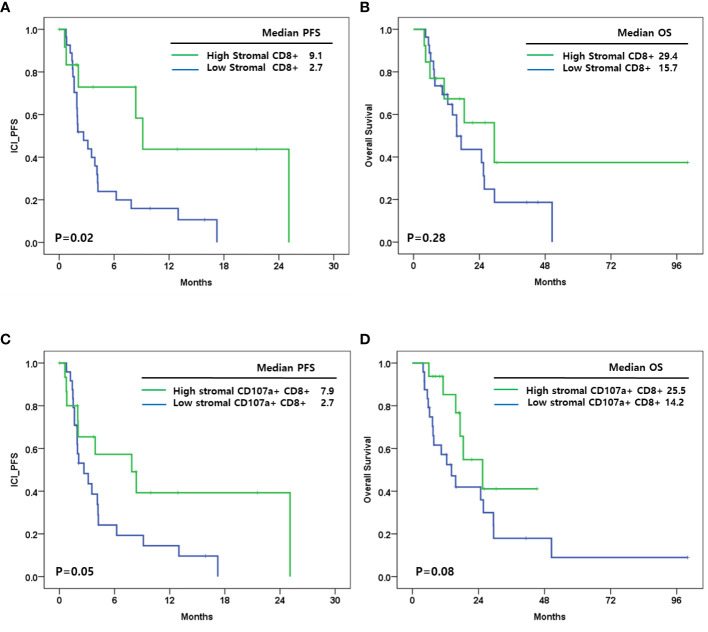
**(A)** Immune checkpoint inhibitor progression free survival(ICI_PFS) according to level of stromal CD8+ cells; **(B)** Overall survival (OS) according to level of stromal CD8+ cells; **(C)** ICI_PFS according to level of stromal CD107a+CD8+ cells; **(D)** OS according to level of stromal CD107a+CD8+ cells.

CD8+ cells were associated with ICI-PFS (**Figure 3A**). A high density of CD8+ cells in stroma had a better ICI-PFS rate compared with a low density of CD8+ cells (9.1 vs. 2.7 months; P=0.02). In OS, there was no difference between high and low density of CD8+ cells in stroma (29.4 vs. 15.7 months; P=0.28) (**Figure 3B**). However, the densities of CD8+ cells in tumor and total area including tumor and stroma were not associated with ICI-PFS and OS.

In addition, CD107a+CD8+ cells in stroma were associated with ICI-PFS (high vs. low: 7.9 vs. 2.7 months; P=0.02) and OS (high vs. low: 25.5 vs. 14.2 months; P=0.08) (**Figures 3C, D**). CD107a+ cells, CD68+CD107a- cells, and CD107a+CD8- cells were not associated with ICI-PFS and OS.

### Prognostic value of PD-L1 and LAG-3 expression

Stromal PD-L1+ and tumor PD-L1+ cell densities were positively correlated (**Supplementary Figure 5A**). Our survival analysis suggested that stromal, tumor, and total PD-L1 expression did not relate to ICI-PFS and OS (**Supplementary Figures 5B, D**). We also evaluated the relationship between PD-L1+LAG-3+ cells and PD-L1+/LAG-3- cells in tumor and stroma. There was no relationship between PD-L1+LAG-3+ cells or PD-L1+LAG-3- cells and either cell distribution or survival outcome (**Supplementary Figures 5E–H**).

LAG3 expression was analyzed in stroma, tumor and total specimen area. In contrast to PD-L1 expression, stromal and tumor LAG-3 expression was not correlated, but stromal LAG-3 expression was directly correlated with total LAG-3 expression (**Supplementary Figures 6A-C**). Additional survival analyses suggested that stromal, tumor, and total LAG-3 expression was not associated with ICI-PFS (**Supplementary Figures 6D–F**).

**Figure 4:**
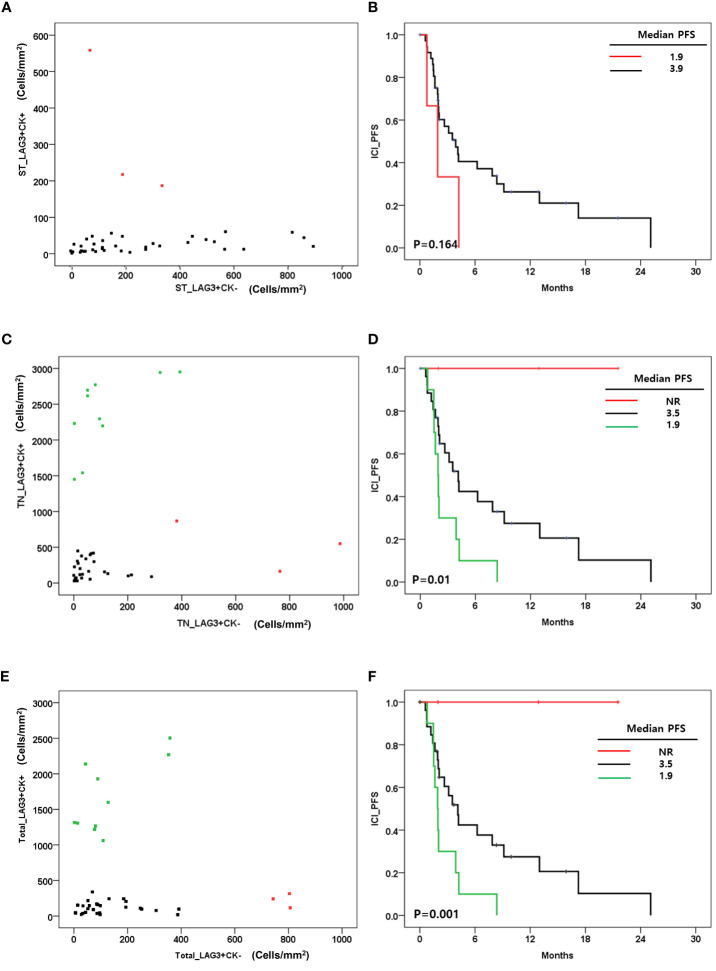
**(A)** Correlation between the level of LAG-3+CK+ cells and LAG-3+CK- cells in stroma (cells/mm2); **(B)** Immune checkpoint inhibitor progression free survival(ICI_PFS) according to level of LAG-3+CK+ cells in stroma(cells/mm2); **(C)** Correlation between the level of LAG-3+CK+ cells and LAG-3+CK- cells in tumor(cells/mm2); **(D)** ICI+PFS according to level of LAG-3+CK+ and LAG-3+CK- cells in tumor(cells/mm2); **(E)** Correlation between the level of LAG-3+CK+ cells and LAG-3+CK- cells in total area(cells/mm2); **(F)** ICI+PFS according to level of LAG-3+CK+ and LAG-3+CK- cells in total area (cells/mm2).

Regarding CK expression, the densities of LAG-3+/CK+ cells and LAG-3+/CK- cells were not correlated in tumor and stroma (**Figures 4A, C**). We evaluated the association between LAG-3+cells and survival outcome regarding CK expression. Stromal LAG-3+/CK+ and LAG-3+/CK- cells were not associated with ICI-PFS (P=0.16) (**Figures 4A, B**). However, LAG-3+ cell distributions in the tumor area impacted ICI-PFS (**Figures 4C, D**). A high density of LAG-3+CK+ cells was associated with shorter ICI-PFS compared with low densities of both LAG-3+CK+ and LAG-3+CK- cells (1.9 vs. 3.5 months). In addition, a high density of LAG-3+CK- cells had a relatively longer ICI-PFS compared with other groups (P=0.01). In terms of total area, the pattern of densities of LAG-3+CK+ cells and LAG-3+CK- cells were similar to those in the tumor area (**Figure 4E**). In addition, ICI-PFS of LAG-3+CK- and LAG-3+CK+ cell densities in the total area was equal to that in the tumor area (**Figure 4F**).

**Figure 5:**
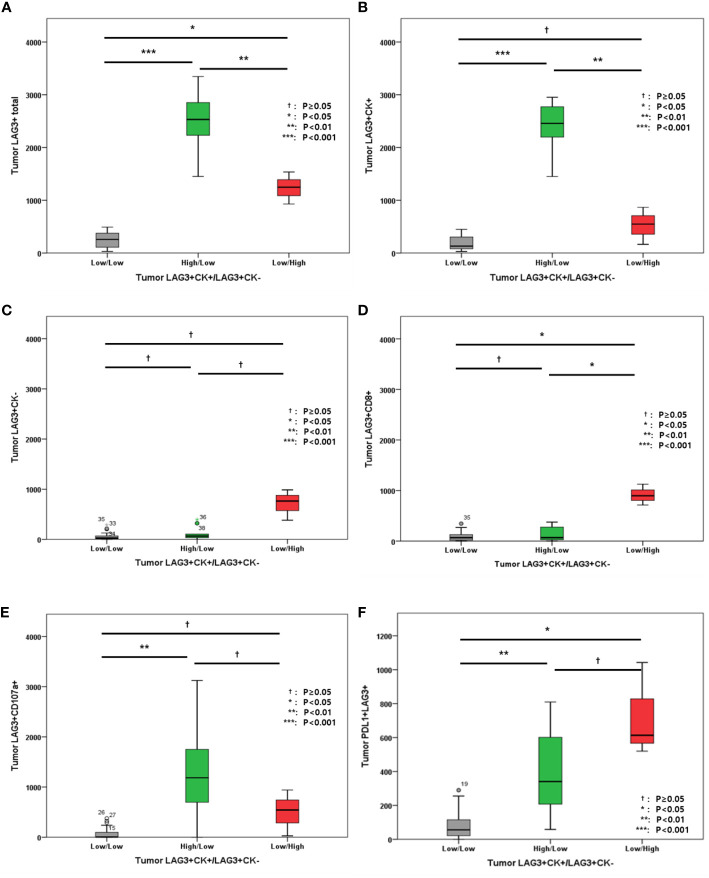
**(A)** Level of tumor LAG3+ cells; **(B)** Level of tumor LAG-3+CK+ cells; **(C)** Level of tumor LAG-3+CK- cells; **(D)** Level of tumor LAG-3+CD8+ cells; **(E)** Level of LAG-3+CD107a+ cells; **(F)** Level of PD-L1+LAG-3+ cells according to tumor LAG-3+CK+/LAG-3+CK- cells.

We also evaluated LAG-3+ cell distribution according to tumor LAG-3+CK+/LAG-3+CK- cell densities. In this analysis, high LAG-3+/CK+ cells were associated with total LAG-3+ cells in tumor (**Figure 5A**), while total LAG-3+CK- cells were associated with total LAG-3+/CD8+ cells (**Figure 5D**). In addition, PD-L1+LAG-3+ cell density was associated with LAG-3+CK+ and LAG-3+CK- cell proportions (**Figure 5F**).

**Figure 6:**
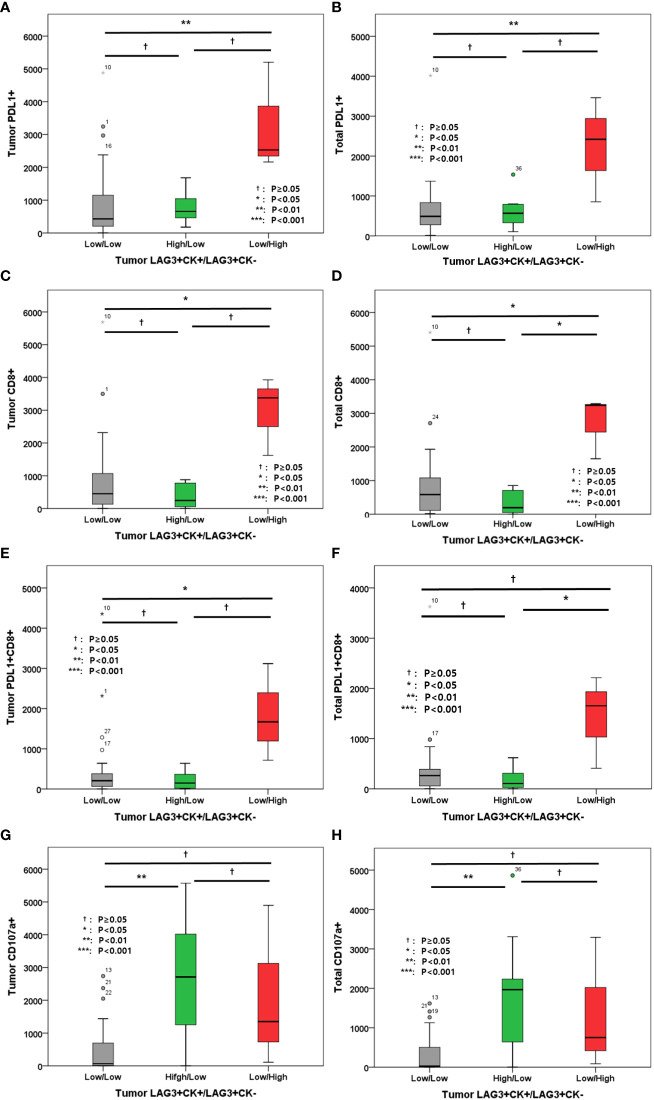
**(A)** Level of tumor PD-L1+ cells; **(B)** Level of total PD-L1+ cells; **(C)** Level of tumor CD8+ cells; **(D)** Level of total CD8+ cells; **(E)** Level of tumor PD-L1+CD8+ cells; **(F)** Level of total PD-L1+CD8+ cells; **(G)** Level of tumor CD107a+ cells; **(H)** Level of total CD107a+ cells according to tumor LAG-3+CK+/LAG-3+CK- cells.

In addition, the associations between other immune markers in tumor and total areas were evaluated (**Figure 6**). High LAG-3+CK- cell density in tumor was associated with high tumor and total PD-L1+ cell densities, high tumor and total CD8+ cell densities, as well as high tumor and total PD-L1+CD8+ cell densities (**Figures 6A–F**). However, tumor and total CD107a+ cell densities were not associated with tumor LAG-3+/CK- cells (**Figures 6G, H**).

**Figure 7:**
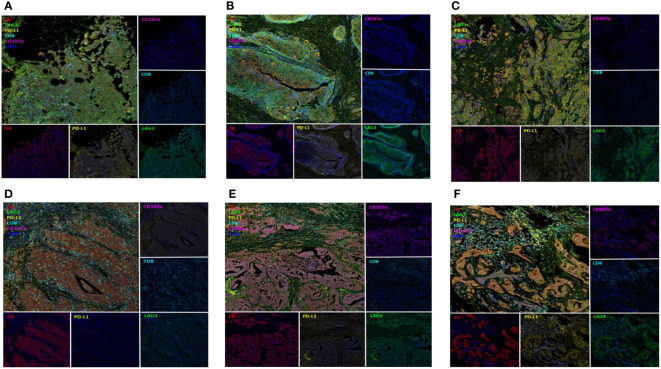
Multiplex Immunohistochemistry according to ICI response; Figures on upper column **(A–C)** presented TNBC with LAG3 expression (green, EPR4392, Abcam) not related to CK+ cells (red, AE1/AE3, DAKO) and having long ICI-PFS whereas figures on lower column **(D–F)** presented TNBC with LAG3 expression on CK+ cells and having short ICI-PFS.

Multi-IHC results for expressed immune-related proteins including LAG-3 are shown in **Figure 7**. The upper three multi-IHC images from mTNBCs had relatively long ICI-PFS, whereas the lower images of mTNBCs show no response to ICI. In the upper three images, LAG-3 expression was not related to CK+ cells, but the lower images suggest that LAG-3 expression was concentrated in CK+ cells.

### Multivariate analysis for ICI-PFS

Multivariate analysis was performed for evaluating prognostic values. In this analysis, LAG-3/CK status in tumor and DRFI were associated with ICI-PFS. High LAG-3+/CK+ and low LAG-3+/CK- cells were also associated with poor ICI-PFS (hazard ratio[HR]: 4.35, 95% confidence interval [CI]: 1.48, 12.77, p=0.028), and TNBCs with under 24 months of DRFI had worse ICI-PFS compared with those had more than 24 months of DRFI (HR:3.66, 95% confidence interval [CI]:1.19, 11.25, p=0.024) (**Table 2**).

**Table 2:** Multivariate analysis of clinico-pathological factors for ICI-PFS (n=37).

Characteristics	Hazard ratio	95% Confidence Interval		P-value
Age				0.073
Younger than 40 vs. older than 40 years	2.33	0.92	5.87	
DRFI				0.024
<24 vs. > 24 months	3.66	1.19	11.25	
Stromal CD8+				0.935
High vs. Low	0.94	0.30	3.04	
Stromal CD107a+CD8+				0.513
High vs. Low	0.72	0.27	1.92	
Total LAG3+				0.028
High LAG3+CK+/Low LAG3+CK- vs. Low LAG3+CK+/Low LAG3+CK-	4.35	1.48	12.77	
Low LAG3+CK+/High LAG3+CK- vs. Low LAG3+CK+/Low LAG3+CK-	NA	NA		

## Discussion

We evaluated the three immune cells CD8+ T cells, natural killer (NK) cells, and macrophages and the two immune checkpoint proteins PD-L1 and LAG-3 in metastatic TNBC treated with the ICIs pembrolizumab and atezolizumab. In this analysis, LAG-3+/CK+ cells were an independent prognostic factor for ICI-PFS, whereas PD-L1 status did not affect ICI-PFS. In addition, stromal CD8+cells and CD8+CD107a+cells were associated with ICI-PFS, but multivariate analysis suggested that these two immune cells had no impact on ICI response.

To date, many cancer patients have been treated with anti-PD-1 or anti-PD-L1 inhibitor combined with cytotoxic chemotherapeutic agents, other ICIs, or targeted agents. In many tumors, PD-L1 status indicates the response to anti-PD-1 and PD-L1 inhibitors according to companion diagnosis associated with clinical trials (15). VENTRA PD-L1 (SP142) assays provide evidence of TNBC patients eligible for atezolizumab treatment and suggest that atezolizumab treatment would be of benefit in TNBC with PD-L1 expression in 1% or more of tumor-infiltrating immune cells (15, 16). Pembrolizumab also could be used depending on PD-L1 status, as represented by the tumor proportion score, which is the percentage of viable tumor cells showing partial or complete membrane staining relative to all viable tumor cells (15, 17). These criteria for ICI treatment were based on clinical trial-proven companion diagnostic assessments (18–20). However, many patients with mTNBCs had short PFS even though mTNBCs were treated by ICIs according to guidelines based on companion diagnostics (4, 5, 21). Indeed, the result of clinical trial of pembrolizumab was effective to TNBC in neoadjuvant setting regardless PD-L1 status (4, 22).

Our study suggests that LAG-3 expression was a poor prognostic marker of ICI response in mTNBC. However, LAG-3+CD8+ cells were suggested as a protective prognostic biomarker in mTNBC with ICI treatment. A previous study of LAG-3 expression in BC suggested that LAG-3 expression was more frequently observed in ER- BC BCs compared with ER+ BCs, and LAG-3 expression in TILs was associated with better prognosis compared with TNBC without LAG-3 expression (23). Another study also suggested that LAG-3 expression guaranteed good prognosis even though it was considered a resistance mechanism for PD-1 axis blockers (24). They also evaluated LAG-3 expression in immune cells but not tumor cells.

These previous studies demonstrated that LAG-3 was expressed on immune cells, though only one study mentioned that tumor-intrinsic LAG-3 protein expression. They evaluated LAG-3 expression in renal cell carcinoma (RCC) cells, and high LAG-3+ RCC was correlated with an elevated level of tumor-infiltrating immune cells. In addition, RCC with high tumor-intrinsic LAG-3 protein expression had worse OS compared with RCC with low LAG-3 expression (25, 26). This result agreed with that of our study, and we suggest that both tumor-intrinsic LAG-3 expression and LAG-3 expression on immune cells are important.

LAG-3 was one immune checkpoint expressed on the cell membrane of NK cells, B cells, TIL, and dendritic cells, and they may have a synergistic interaction with PD-1/PD-L1 as immune checkpoints (27). This was an inhibitory regulator that control signaling pathways of T cells and antigen presenting cells and LAG-3 signaling pathway inhibited early events in primary activation of human CD4 and CD8 T cells (28, 29). In addition, tumor microenvironment with PD-1 and LAG-3 co-expression mediated the immune escape effect of tumor cells (30). Given the resistance mechanism of ICIs was mediated by additional immune checkpoints, LAG-3 played one of escape mechanism of PD-1/PD-L1 inhibitors and induced ICI-resistance (31, 32). Therefore, LAG-3 inhibition was the mechanism to overcome PD-1/PD-L1 inhibitor resistance, and pre-clinical studies have suggested that dual knockdown of LAG-3 and PD-1 increases survival in mice with transplanted tumors (33). In addition, recent clinical trials of LAG-3/PD-1 combination therapy for melanoma had positive outcome (13). To date, clinical trials of LAG-3/PD-1 combination and LAG-3/PD-1 bispecific antibody in solid tumors have progressed, and we are anticipating the results of these clinical trials.

Our cohort consisting of mTNBCs had poor prognosis. Up to 70% of patients underwent distant recurrence of TNBC in 24 months of initial BC diagnosis, and 12 of 37 patients experienced distant BC recurrences in 12 months. This indicated that such patients rarely respond to cytotoxic chemotherapy in a metastatic setting and have short OS. Therefore, our study suggested that the role of LAG-3 expression in mTNBCs with poor prognosis is related to unmet treatment needs.

In our study, we showed that BC with high LAG-3+CK+ cell numbers had worse treatment outcomes with PD-L1/PD-1 inhibitor, whereas BC with high LAG-3+CK- cell numbers had better outcomes compared to those with both low LAG-3+CK+ and low LAG-3+CK- cells. In addition, tumor and total CD8+ cell densities were highest in BC high-density LAG-3+CK- cells compared with other BC groups. This had in common with tumor infiltrating tumor (TIL)s a role as an indicator of good survival outcomes in BC. Moreover, BC with high LAG-3+CK-/low LAG-3+CK+ cell densities had high PD-L1+ and PD-L1+CD8+ cell densities. Therefore, these PD-L1+ immune cell infiltrations were positively associated with response to PD-L1/PD-1 inhibitors. Overall, tumor-intrinsic LAG-3+ expression indicates poor response to PD-1/PD-L1 inhibitors in mTNBCs, but LAG-3+ in CK- cells, consisting of immune cells including PD-L1+ cells, results in the opposite response for PD-1/PD-L1 inhibitors. This also could explain why the results of clinical trials for TNBCs with PD-L1/PD-1 inhibitors were controversial according to PD-L1 state.

Our study had some limitations. First, we only evaluated 37 metastatic TNBC tissues of 40 patients who treated with PD-1/PD-L1 inhibitor. In addition, we did not use immune RECIST (iRECIST) criteria to evaluate tumor response treated with ICIs (34). However, recent meta-analysis suggested that response evaluation with iRECIST did not differ to that with RECISIT 1.1 on response-related endpoint including ORR. Therefore, our evaluation of tumor response might be sufficient even though we did not use iRECIST criteria in this study.

Despite the small numbers of evaluated tissues, our study suggests that tumor-intrinsic LAG-3 expression is the resistance mechanism toward PD-1/PD-L1 inhibitors in mTNBCs. Multivariate analysis also suggested that LAG-3 expression in tumor cells was an independent predictive biomarker. A further large-scale, translational study for LAG-3 expression in TNBC is warranted to confirm the role of tumor-intrinsic LAG-3 expression in mTNBC treated with PD-1/PD-L1 inhibitors. In addition, our study could be used to design clinical trials of LAG-3 inhibitor, a new, promising ICI for TNBCs.

## Data availability statement

The original contributions presented in the study are included in the article/**Supplementary Material**. Further inquiries can be directed to the corresponding authors.

## Ethics statement

The studies involving human participants were reviewed and approved by Institutional Review Board (IRB) of Samsung Medical Center, Seoul, Korea. The patients/participants provided their written informed consent to participate in this study.

## Author contributions

Conceived and supervised study, K-MK and Y-HI. Investigation and acquisition of data (sample collection and processing and collection of patient information), J-YK, EC, YP, JA, and Y-HI. Investigation (contributed to multiplex data), J-YK, JK, and K-MK. Investigation (pathology review), EC and K-MK. Data analysis, data integration, and interpretation, J-YK. Writing manuscript, J-YK. Reviewing and editing manuscript, all authors. All authors contributed to the article and approved the submitted version.

## Supplementary material

Supplementary Table 1 Cell counts and cell densities of multiplex immunohistochemistry
