# Supplementary material for: Lymphocyte-activating gene 3 expression in tumor cells predicts immune checkpoint inhibitor response in triple negative breast cancer
Source: Front Oncol. 2023 Mar 16;13:1146934. doi: 10.3389/fonc.2023.1146934 (PMC10060989; doi:10.3389/fonc.2023.1146934)

Supplementary Figure 1. Consort diagram

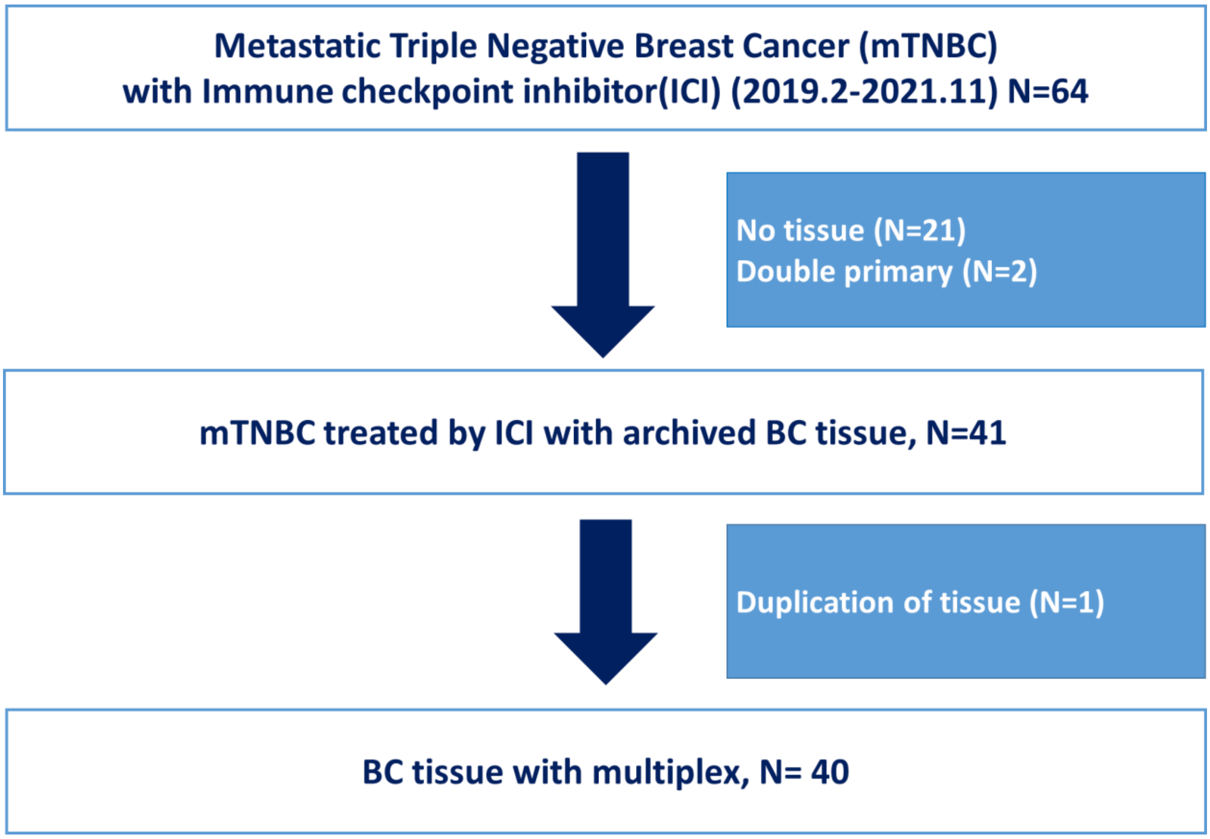

Supplementary Figure 2A. Progression Free Survival (PFS); 2B. Overall Survival (OS); 2C. PFS regarding immune check point inhibitors (ICIs); pembrolizumab and atezolizumab; 2D. OS regarding ICIs; 2E. PFS regarding ICI treatment lines

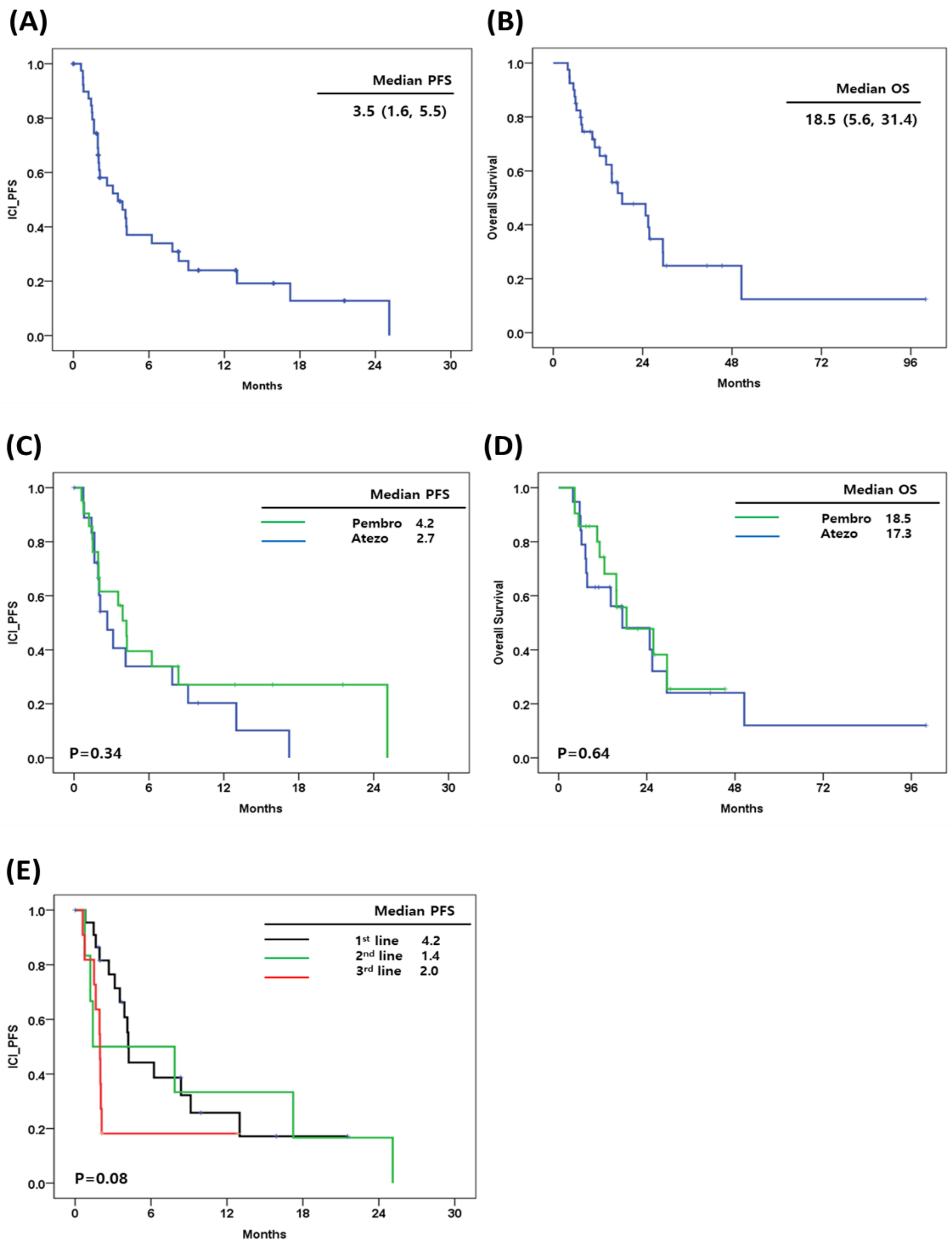

Supplementary Figure 3. Level of LAG-3 expression according to stroma, tumor and total area

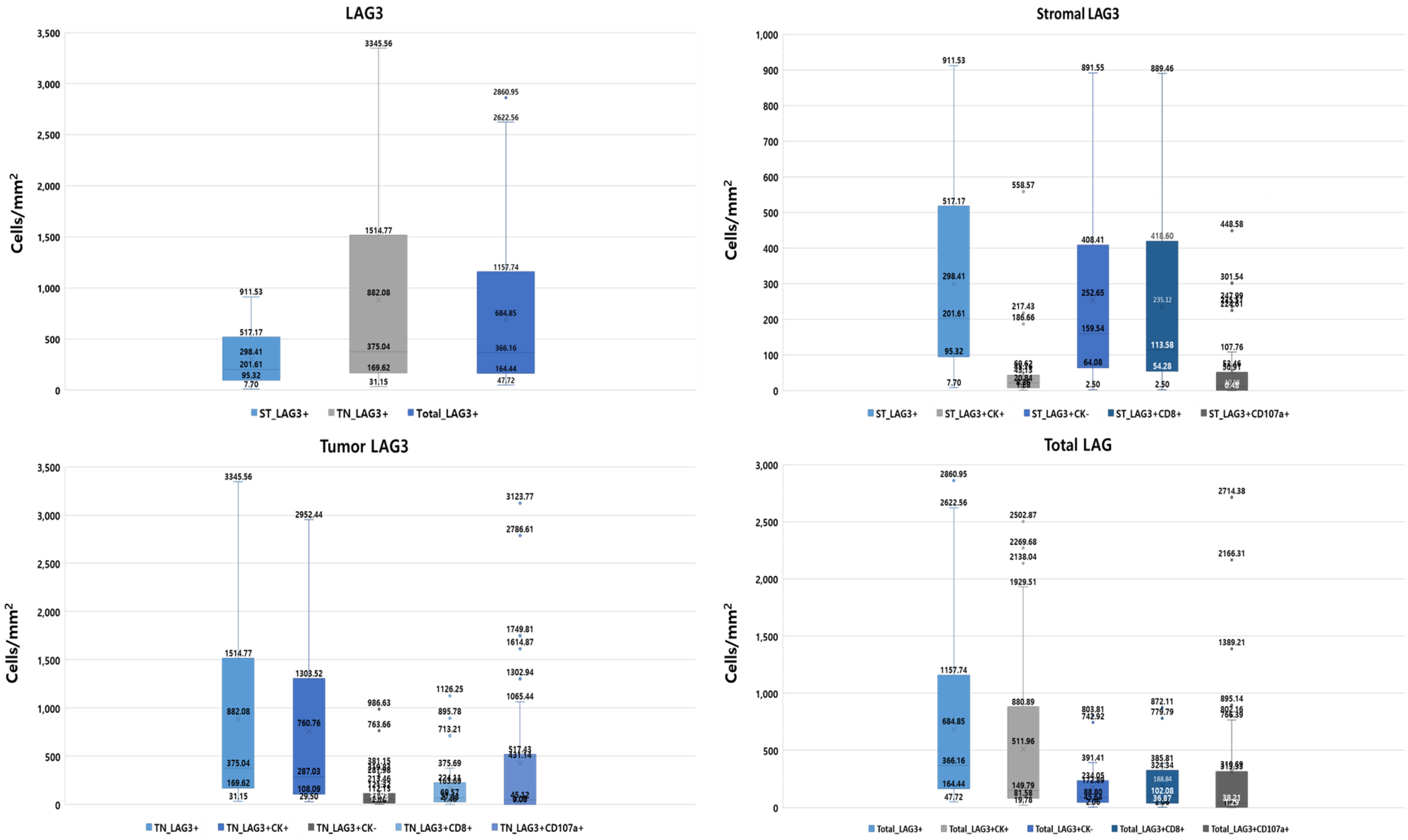

Supplementary Figure 4A. Correlation between level of CD8+ cells between stroma and tumor ; 4B. Correlation between level of CD107a+ cells between stroma and tumor; 4C. Correlation between level of CD107a+CD8+ cells between stroma and tumor

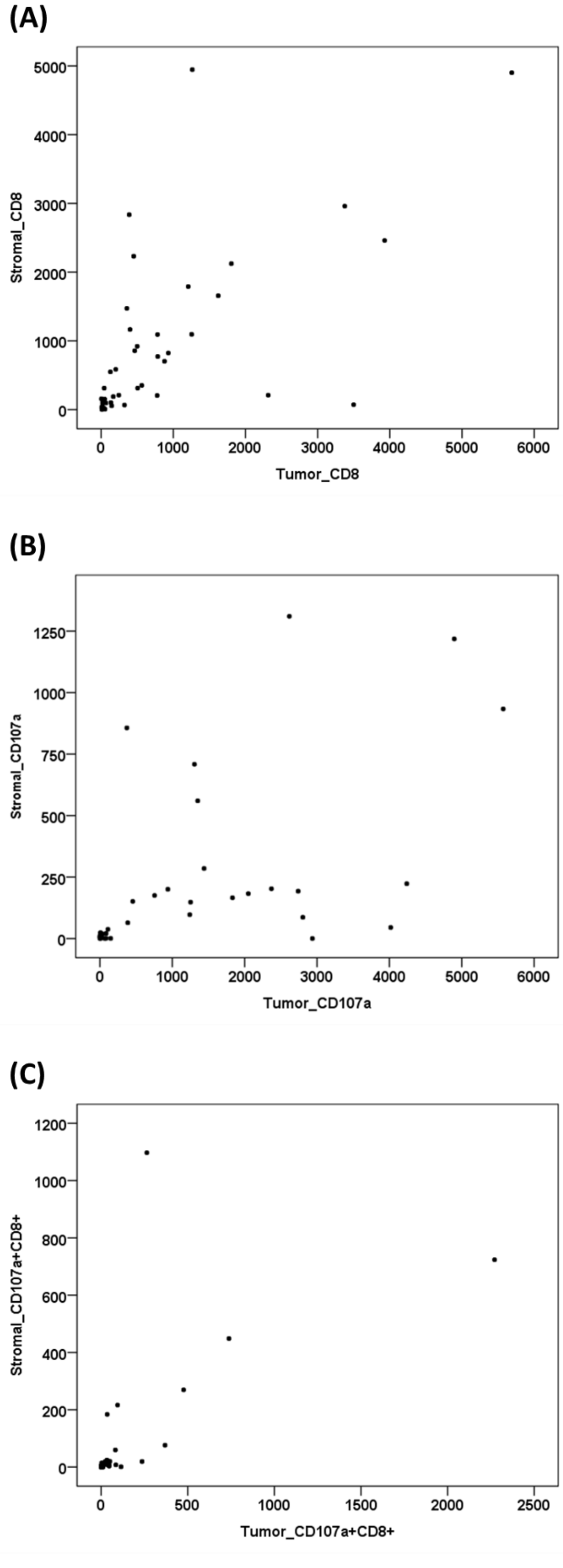

Supplementary Figure 5A. Correlation between level of PD-L1+ cells between stroma and tumor ; 5B. Immune checkpoint inhibitor related progression free survival (ICI-PFS) according to PD-L1+ cell status; 5C. ICI-PFS according to stromal PD-L1 cell status; 5D. ICI-PFS according to tumor PD-L1 cell status; 5E. Correlation between level of stromal PD-L1+LAG3- cells and PD-L1+LAG3+ cells; 5F. Correlation between level of tumor PD-L1+LAG3- cells and PD-L1+LAG3+ cells; 5G. Correlation between level of total PD-L1+LAG3- cells and PD-L1+LAG3+ cells; 5H. ICI-PFS according to the status of total PD-L1+LAG3- cells and PD-L1+LAG3+ cells.

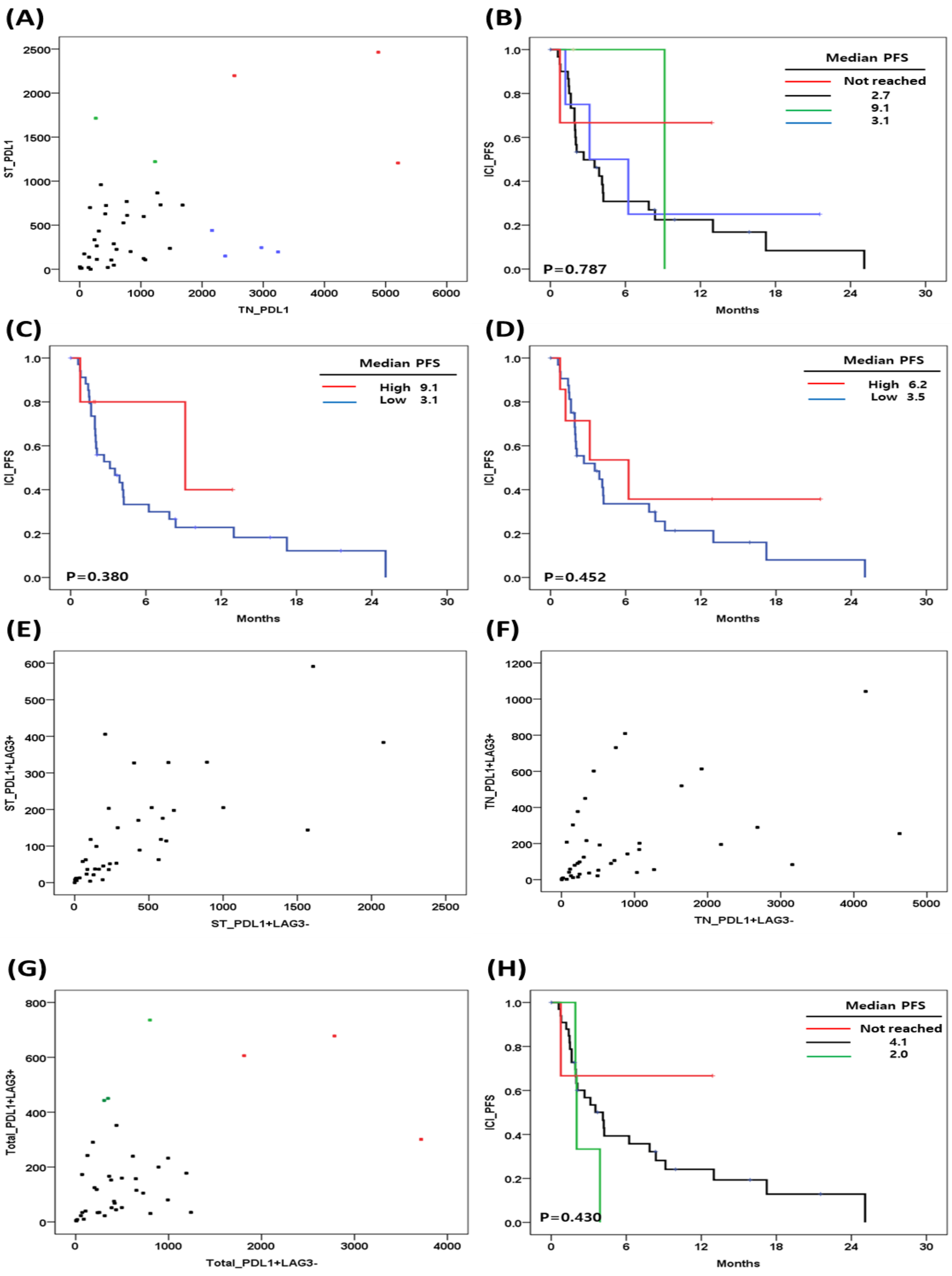

Supplementary Figure 6A. Level of LAG3+ cells between stroma and tumor; 6B. Level of LAG3+ cells between stroma and total area; 6C. Level of LAG3+ cells between tumor and total area; 6D. Immune checkpoint inhibitor progression free survival (ICI_PFS) according to level of LAG3+ cells in stroma; 6E. ICI_PFS according to level of LAG3+ cells in tumor; 6F. ICI_PFS according to level of LAG3+ cells in total area

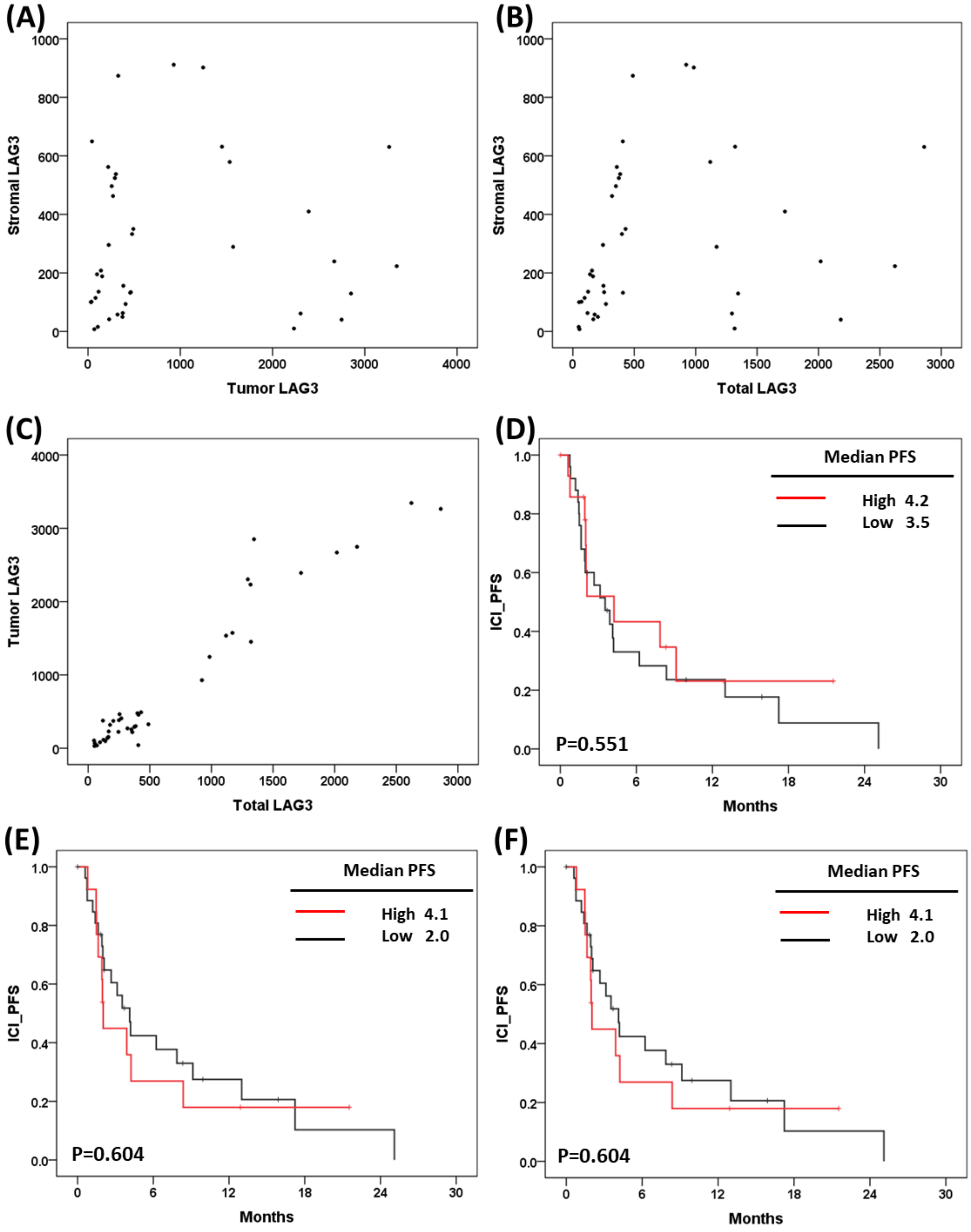

Supplement: Supplementary file 1 [file Image_1.pdf]
